# Influence of Physical Fitness, Anthropometric Profile, and Biological Maturation on Technical Performance and Enjoyment of Untrained Children Who Participate in Continuous and Fractional Small-Sided Games

**DOI:** 10.3390/children9111730

**Published:** 2022-11-10

**Authors:** Nicolás Gómez-Álvarez, Hernán Costa Luengo, Leonardo Alarcón Lamilla, Kamir Barraza Álvarez, Valeria Espinoza Salinas, Yesenia Olate-Pasten, Cristian Godoy-Tapia, Gustavo Pavez-Adasme, Felipe Hermosilla-Palma

**Affiliations:** 1 Physical Activity, Health and Education Research Group (AFSYE), Physical Education Pedagogy, Universidad Adventista de Chile, Camino a Tanilvoro Km. 12, Chillán 3780000, Chile; 2 Centre for Research, Education, Innovation, and Intervention in Sport (CIFI2D), Faculty of Sport, University of Porto (FADEUP), Rua Dr. Plácido da Costa 91, 4200-450 Porto, Portugal; 3 Pedagogía en Educación Física, Facultad de Educación, Universidad Autónoma de Chile, Talca 3460000, Chile

**Keywords:** physical education, team sports, sports education, childhood

## Abstract

The objective is to determine the relationship between physical fitness, anthropometric measures, and biological maturation as they relate to technical performance in small-sided games (SSGs) of continuous and fractioned regimes. Methodology: A crossover-design study in which 12 children participated in two regimens of SSG (continuous and fractional). At the beginning of the study, all children were evaluated using physical fitness tests (horizontal jump test, vertical jump, cardiorespiratory fitness, and agility), anthropometric profile (weight, height, Body Mass Index (BMI), and waist circumference (WC)), and biological maturation (peak years of growth velocity). All sessions were recorded and analyzed with the Performance Assessment in Team Sports instrument, and at the end of each game each child was asked to answer a scale of enjoyment for physical activity. Results: The results of the paired samples t-test showed no significant differences in the measures of technical performance and perceived enjoyment for the continuous and fractional regimens of SSGs (*p* > 0.05). The correlation results showed that technical performance in the continuous and fractional regimes was related to agility, horizontal jump, and height, while biological maturation was only related to technical performance in the fractional regimen of SSGs. Perceived enjoyment showed a negative relationship with weight, height, BMI, and WC. Conclusion: The fractional and continuous regimens of SSGs implemented in this study induced similar technical demands and enjoyment. Furthermore, the results suggest that physical fitness, anthropometric profile, and biological maturation may influence the technical performance and enjoyment of SSGs.

## 1. Introduction

The World Health Organization recommends that children and adolescents should engage in at least 60 min a day of moderate to vigorous physical activity (MVPA) and should incorporate at least three days a week of exercises that strengthen muscles and bones [1]. However, the prevalence of physical inactivity is estimated to be over 80% in both sexes worldwide, while in Chile the figures reach 84.2% in boys and 91.2% in girls [2]. Given the need to establish strategies that promote physical activity, it has been identified that interventions at the school level could be effective in promoting physical activity [3].

In particular, schools’ physical education (PE) classes could offer a great opportunity to favor the development of physical fitness and motor skills in children and adolescents [4], encouraging them not only to comply with PA recommendations, [5] but also providing children and adolescents with tools with which they can continue with sports activities outside of a school context [6]. Unfortunately, it has been found that PE is not able to provide students with the motivation to create habits of practicing sports or other physical activities outside school hours [7]. It is now proposed that interventions should aim to develop physical literacy, understood as the motivation, confidence, physical competence, knowledge, and understanding to value and engage in lifelong physical activity [8].

The implementation of teaching models appropriate to the needs of students and the context can be key to the development of physical literacy [7]. Traditionally, the PE classroom has been characterized by models that encourage a controlled, disciplined, and safe classroom organization [9], restricting not only playfulness but also motivation and autonomous participation [10]. However, currently in PE, different practice-based pedagogical models have been established that could favor student motivation and participation, for example, teaching for understanding, non-linear pedagogy, and sports pedagogy, which have been shown to be positive alternatives that are also accepted by teachers [11,12,13].

In this sense, small-sided games (SSGs) are widely used in pedagogical models that favor the practice, offering the integration of skills in contextualized situations [7,11] in which different variables that affect the dynamics of the game are manipulated (for example, the number of players, court dimensions, rules, duration, and coach interventions) [14,15]. Evidence shows that SSGs are a powerful methodological resource that could favor the development of technical and tactical skills in various sports modalities [14,16]; unfortunately, much of the research has been conducted on young athletes and evidence in the untrained population is limited.

Previously, different systematic reviews have proposed that regimen type or participant characteristics should be considered when planning an SSG for desired responses [14,15,16]. However, the effect of SSG regimen type finds inconsistent results [15], although some results suggest that participants with low cardiorespiratory fitness would have lower technical performance in continuous regimens [14]. Similarly, the influence of participant characteristics on SSG outcomes has limited evidence [14,16], and differences between age, maturity status, or physical fitness may be mediated by technical skills possessed by participants [16].

Considering the increased use of SSGs in non-competitive contexts and the limited evidence about the effect that regimen type or individual characteristics might have on technical performance or enjoyment during SSGs, this research aimed to determine the relationship between physical fitness, anthropometric measures, and biological maturation with technical performance and perceived enjoyment in untrained children during continuous- and fractional-regimen small-sided games.

## 2. Materials and Methods

**Figure 1:**
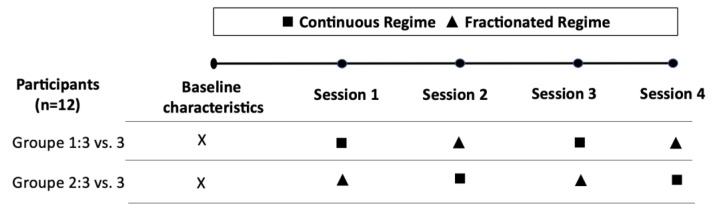
Experimental design.

A crossover experimental design study was carried out in which 12 children participated in 4 sessions of small-sided games, two sessions of fractional regimens (4 bouts of 4 min, with 4 periods of rest), and two continuous regimens (1 match of 16 min). Participants were assigned to a team of 3 participants and always faced the same opposing team in each of the 4 experimental sessions. Each team played only 1 SSG regimen per session and they were performed alternately (see Figure 1).

The research was approved by the ethics committee of the Adventist University of Chile (Nº 2021-17). All parents and children signed their respective informed consent or assent. All procedures were carried out following the principles described in the Helsinki declaration for human studies [17].

Participants

Twelve untrained children (11.5 ± 1.33 years) were invited to participate in the study. All children were from a recreational soccer school that trained once a week and resumed activities after 16 months of suspension of activities due to confinement during the COVID-19 pandemic. Inclusion criteria were: (i) boys between 11 and 14 years of age; (ii) informed consent and assent signed by each guardian responsible for the schoolchild; (iii) no participation in structured training during the last 6 months; and (iv) health compatible with the practice of physical activity. Exclusion criteria: (i) reporting an injury or severe muscle pain during the data collection sessions; (ii) history of musculoskeletal injuries in the last 6 months; (iii) motor or intellectual disability that does not allow him/her to perform team sports; or (iv) failure to comply with instructions during evaluations or soccer sessions.

Procedures

A member of the research team informed the participants and their legal guardians of the objectives of the research project, the days, the schedule, and the place where the sessions would take place. Data collection was carried out for 3 weeks. In the first week anthropometric and sociodemographic evaluations were performed, followed by jumping tests: horizontal and vertical jump, T-test agility test, and YO-YO intermittent recovery test. In the second and third weeks, the experimental sessions were carried out, allowing a 24 h recovery period between each session. During the SSG sessions, each participant wore a Polar H10 brand heart band [18] connected to the SPARKvue application to record heart rate (HR) data. All sessions were recorded using two sports cameras (GoPro Hero 8 Black and DJI-Osmo Action) in a similar way to previous research [19], to later analyze each match under individual performance assessment [20]. Before the start of the match, all participants performed a warm-up guided by a member of the research group that lasted approximately 7 min, and after each match, they completed the Physical Activity Enjoyment Scale (PACES).

Small-sided games Intervention (SSG)

All experimental sessions began with a targeted, staged warm-up. The SSGs consisted of 3 vs. 3 formats in two types of regimes; the first was the “continuous regime” which was a 16 min one-half match, while the other mode of play was the “fractional regime”, which was composed of 4 halves, lasting 4 min each, with a 2 min break for each half. At both ends of the court there were members of the research groups, who had balls available for each kick of the participants, to provide greater fluidity to the game. The rules used for these reduced games were as follows: the goal is valid when all participants pass half court; throw-ins and corner kicks were performed with the foot; and after each goal, the game was resumed at half court. As for the playing surface, the area was delimited with cones, measuring 17 m long by 9 m wide, while the goal was 78 cm high by 2.9 m wide.

Instruments

Anthropometric measurements: Body mass, height, sitting height, and waist circumference were measured with standardized protocols using shorts and polo shirt (Seca^®^ scale, model 803; Seca^®^ stadiometer, model 213; and CESCORF^®^ anthropometric tape). Body Mass Index (BMI) and years at peak growth velocity (APHV) were calculated [21].

Physical fitness measurements: cardiorespiratory fitness, muscular fitness, and agility tests were performed as follows:

Cardiorespiratory fitness measurements: These were evaluated by the YO-YO Intermittent Recovery Test (YYIRT), according to the protocol proposed by Krustrup et al. (2003) [22]. The test consisted of running a distance of 20 m at an increasing speed, starting at 8.5 km/h, with increments of 0.5 km/h every 1 min, until exhaustion. The test ended when the child failed to complete the distance between beeps twice.

Muscular fitness measurements: These were evaluated using a vertical jump with countermovement (CMJ) and a horizontal jump (SH), performing three attempts each. The CMJ was evaluated with a contact mat (Chronojump^®^ Boscosystem, Barcelona, Spain) and participants were asked to lower their center of gravity to a self-selected depth and then perform a vertical jump, always with their hands on their hips. The same verbal prompts were delivered before each attempt, “jump as high as you can in 3, 2, 1 go” [23]. The SH was performed according to the protocol described by Castro-Piñero et al. (2010) [24]. Each participant stood behind a starting line with feet together and made a forward jump reaching as far as possible. With an inextensible tape measure accurate to 0.1 cm, the distance from the starting line to the heel of the foot that landed closest to this line was measured.

Agility test: To measure agility, each participant performed three attempts of the agility test T-test (TT) [25]. The TT consists of performing a linear sprint forward for 9.14 m, lateral to the left for 4.57 m, lateral to the right for 9.14 m, lateral to the left for 4.57 m, and linear backward for 9.14 m. The data were recorded using a system that allows for the measurement of agility. Data were recorded using a photocell system (Chronojump^®^ Boscosystem, Barcelona, Spain).

Measures of performance and enjoyment during the SSG: Performance during the SSG was evaluated by observing video records using the Performance Assessment in Team Sports instrument [20]. In addition, at the end of each game, participants completed the Physical Activity Enjoyment Scale (PACES) [26].

Individual performance assessment: Each player was observed during the games, and various actions that occurred during each game were recorded based on the Performance Assessment in Team Sports instrument [20]. The actions observed were: (1) conquering the ball (CB), which consists of the interception of the ball, theft of the ball, or recovery of the ball after a failed shot at goal, resulting in the effective possession of the ball; (2) receiving the ball (RB) from a teammate, obtaining possession of it without losing it immediately; (3) playing a neutral ball (NB), which corresponds to a pass whose objective is to maintain possession of the ball without intending to generate an attack on the opposing team; (4) losing the ball (LB), according to which a player is considered to have lost the ball when possession is interrupted by the opponent’s action or by the player’s own error; (5) playing an offensive ball (OB), which corresponds to a pass to a teammate that exerts pressure on the other team and, most often, leads to a shot on goal; and(6) executing a successful shot (SS), which is when the player scores or the team retains possession of the ball. With these indicators, the technical performance indexes were calculated: Volume of Play (VP; CB + RB), Efficiency Index (EI; (OB + SS)/(10 + LB)), and General Performance (GP; (VP/2) + (EI × 10)). The observation process was carried out by researchers who were previously trained.

Enjoyment Scale (PACES): At the end of each SSG, participants completed the Physical Activity Enjoyment Scale (PACES) [26], which consists of 16 items rated from 1 to 5, with 1 being “strongly disagree” and 5 being “strongly agree”. For example, positive items referred to statements such as “I enjoy it”, “I find it pleasurable”, and negative items had statements such as “I dislike it” or “It’s no fun at all.”

Statistical procedures

Results are presented as means and standard deviation (SD) for descriptive data. The normality analysis of data distribution was tested with the Shapiro–Wilk test. The results of the reduced sets with continuous (2) and fractional (2) regimes were averaged for comparison and correlations.

Differences between continuous and fractional regimes for measures of game performance, physiological responses, and perceptual responses were performed with paired samples t-tests. In addition, Cohen’s d effect size with 95%CI was calculated and classified as trivial (<0.2), small (0.2–0.6), moderate (0.6–1.2), or large (>1.2).

Pearson product-moment correlation coefficients tested the relationships between performance during reduced play with anthropometric and fitness variables. The magnitude of the correlation is reported for all variables and supplemented as 95% confidence intervals and r2 when a significant correlation was found between variables. The magnitude of the correlation between variables was assessed with the following thresholds: <0.1, trivial; 0.1–0.3, small; 0.3–0.5, moderate; 0.5–0.7, large; 0.7–0.9, very large; and >0.9, near perfect [27]. All analyses were performed using JAMOVI statistical software (version 2.2), with a significance level of *p* < 0.05.

## 3. Results

The anthropometric and physical fitness characteristics of the study participants are presented in Table 1.

The results for the measures of technical performance, perceived enjoyment, and physiological response for the continuous and fractional regimens are presented in Table 2. The results of the paired samples t-test showed no significant differences in any of the performance measures evaluated. In addition, the effect size for the difference between continuous vs. fractional SSG was small for balls conquered (ES: −0.38 95%Cl (−0.95–0.22)) and trivial for the rest of the measures. Differences between continuous and fractional regimens showed no significant differences for PACES or heart rate with small effect sizes for PACES differences (ES:0.59, 95%Cl: (−0.06–1.23) and trivial effect sizes for heart rate response.

The correlation coefficients between technical performance and enjoyment during SSG with anthropometric and fitness measures are presented in Table 3. Anthropometric measures did not show a significant correlation in technical performance during continuous SSGs. However, in fractional SSGs, height showed a positive correlation with the efficiency index (R = 0.58, 95%CI. [0.01:0. 87]; R2 = 0.34; *p* = 0.04), while years to APHV was positively correlated with successful shots (R = 0.64, 95%CI [0.11:0.89]; R2 = 0.41; *p* = 0.02) and efficiency index (R = 0.71, 95%CI [0.24:0.91]; R2 = 0.50; *p* = 0.01).

In addition, a relationship was found between PACES scores, during continuous and fractional SSGs, with height (R = −0.725, 95%CI (−0.22:−0.92); R2 = 0.53; *p* = 0.01 and R = −0.75, 95%CI (−0.27:−0.93); R2 = 0.56; *p* = 0.008), weight (R = −0.82, 95%CI (−0.44:−0.95); R2 = 0.67; *p* = 0.002 and R = 0.84, 95%CI (−0.48:−0.96); R2 = 0.71; *p* = 0.001), body mass index (R = −0.78, 95%CI (−0.34:−0.94); R2 = 0.61; *p* = 0.005 and R = −0.82, 95%CI (−0.44:−0.95); R2 = 0.67; *p* = 0.002), and waist circumference (R = −0.82, 95%CI (−0.44:−0.95); R2 = 0.67; *p* = 0.002 and R = −0.90, 95%CI (−0.67:0.98); R2 = 0.81; *p* < 0.001).

The results of the relationship between physical fitness and continuous SSGs showed a positive correlation between horizontal jumping and offensive balls (R = 0.60, 95%CI (0.05:0.88); R2 = 0.36; *p* = 0.036), efficiency index (R = 0.67, 95%CI (0.15:0.89); R2 = 0.45; *p* = 0.02), and overall performance (R = 0.65, 95%CI (0.13:0.89); R2 = 0.42; *p* = 0.02). Furthermore, a negative correlation was found between agility test execution time with the number of successful shots (R = −0.63, 95%CI (0.08:0.88); R2 = 0.40; *p* = 0.03) and the efficiency index (R = −0.63, 95%CI (0.09:0.88); R2 = 0.40; *p* = 0.03).

In fractional SSGs, the horizontal jump score correlated with successful shots (R = 0.60, 95%CI (0.05:0.88); R2 = 0.36; *p* = 0.04) and the efficacy index (R = 0.60, 95%CI [0.04:0.87); R2 = 0. 36; *p* = 0.04), while the results in the agility test correlated with balls conquered (R = −0.64, 95%CI (−0.10: −0.89); R2 = 0.41; *p* = 0.03), balls received (R = −0.69, CI95% (−0. 19:−0.90); R2 = 0.40; *p* = 0.01), offensive balls (R = −0.78, 95%CI (−0.37:−0.94); R2 = 0.62; *p* = 0.003), successful shots (R = −0.73, 95%CI (−0.26:−0.92); R2 = 0.53; *p* = 0.007), volume of play (R = −0. 72, 95%CI (−0.25:−0.92); R2 = 0.41; *p* = 0.008), efficiency index (R = −0.68, 95%CI (−0.17:−0.90); R2 = 0.46; *p* = 0.02), and overall performance (R = −0.82, 95%CI (−0.45:−0.95); R2 = 0.67; *p* = 0.001).

## 4. Discussion

The purpose of this study was to determine the relationship between physical fitness, anthropometric measures, and biological maturation as they relate to performance in small-sided games of continuous and fractional regimens in untrained children. Results showed no differences between regimens with respect to technical performance or perceived enjoyment during SSGs. Correlation analysis found that height and APHV were related to efficiency rate and successful shots in fractional SSGs, but not in continuous SSGs. Physical fitness measures, mainly horizontal jump and agility, correlated with technical performance measures in both SSG modalities. In addition, perceived enjoyment during continuous and fractional SSGs was related to weight, height, BMI, and waist circumference.

Our study implemented SSGs in continuous and fractional regimens, without finding significant differences in technical performance or perceived enjoyment during the game. Previously, systematic reviews have reported a relationship between physical fatigue and technical performance [28]; therefore, it might be concluded that regimens that generate a higher workload could negatively affect performance. Studies that have compared the two types of regimens have found that fractional SSGs generate a greater internal and external load [29,30]. The results of this study showed no difference in heart rate response, considered an indicator of internal load, which could explain a similar technical performance in both regimens. These inconsistencies have been previously described in systematic reviews, and the evidence has failed to reach a conclusion regarding the effect of regime type on technical performance [14,16].

Previous research has reported that technical performance during SSGs could be influenced by the individual characteristics of the participants [14,31]. The results of this study showed that anthropometric characteristics and physical fitness are related to technical performance. Moreover, these variables influenced the fractional regimen more than the continuous regimen. Specifically, only height influenced balls conquered in the continuous regime, while height and APHV influenced the efficiency rate and successful shots. Similarly, horizontal jump and agility influenced technical performance in more variables in the fractional regime than in the continuous regime.

In trained populations, it has been found that locomotor demands can vary according to the regime or duration of the series [15,32]. Particularly, intermittent regimens would be associated with a greater distance covered [33], which could represent a greater number of actions involving changes of direction [34]. Therefore, a better performance in the agility test would represent a favorable condition to face the locomotor demands that are more prevalent in fractional games, and so may result in higher technical performance. Additionally, performance in horizontal jumping has been related to performance in activities with a change of direction [35], which could reflect a greater capacity for force production in the horizontal direction.

Our results showed that biological maturation measured by the APHV had a moderate relationship with technical performance only for the fractional regime but not in the continuous regime, showing that those with a higher maturational status had higher performance in successful shots and in the efficiency index. Interestingly, technical performance during the fractional regime showed a greater influence of performance in the agility test, a skill that is related to maturational status [36]. On the other hand, contradictory results have been reported in the literature, for example, Moreira et al. (2017), found that hormonal status, sexual maturation, anthropometric profile, and physical performance influence technical skills in elite youth players [31], while da Silva et al. (2011), found no relationship between sexual maturation and technical performance in youth at similar ages. Since our results were also contradictory, it seems necessary to analyze the interaction between biological maturation and physical fitness on technical performance, especially in untrained populations in which we can find greater interindividual differences [37].

The SSG is proposed as a resource associated with a high level of enjoyment, with the potential to be applied in different contexts, such as school, sports, or health promotion programs [14,38]. In addition, the enjoyment of physical activity is considered key in the promotion of active lifestyles [6,39]. The results of this study showed that perceived enjoyment of both SSG regimens had a large inverse relationship with anthropometric variables but not to physical fitness or maturity status. This seems to be relevant, especially because in recent years SSGs have been proposed as a strategy for the prevention and treatment of obesity [38,40].

The SSG is considered a methodological resource for teaching sports [14], and different reviews have been identified that analyze their technical and physical dimensions [14,15]. However, only a limited number of studies have analyzed the acute effects in a school or untrained population. Mainly, the analyses have emphasized physiological, psychological, and only a few on technical aspects [40,41,42,43]. The results of the present research highlight the importance of considering the development of physical skills such as agility and horizontal jump in the technical performance that could be achieved, and that the anthropometric measures of weight, BMI, and WC are inversely related to the enjoyment of the SSG.

The results of this research are important to understand how untrained children and adolescents respond to SSGs, and thus, to understand the different variables that determine the performance and experience associated with SSGs in contexts such as school, recreational sports workshops, or health programs that use this type of resource. However, it should be taken into consideration that the sample size is small, so future research should cover a larger sample size to achieve a more precise understanding of the influence of physical fitness and anthropometric variables on the technical performance in and enjoyment of SSGs.

## 5. Conclusions

The fractional- and continuous-regimen SSGs implemented in this study induced similar technical demands and enjoyment. In addition, the results showed that better results in the agility test, horizontal jump, and the height of the participants were related to better technical performance, but only the anthropometric profile constituted by height, weight, Body Mass Index, and waist circumference was related to enjoyment. Biological maturation could be related to technical performance in fractional regimes. Therefore, considering the importance of offering enriching sports experiences, teachers or coaches should use continuous or fractional reduced games, considering the anthropometric profile and physical fitness of the participants in the planning of SSGs intended for untrained children and adolescents.

## Figures and Tables

**Table 1:** Characteristics of the participants.

Variables	Mean ± SD	Min–Max
Anthropometrics		
Weight, kg	42.99 ± 12.0	27.6–66.9
Height, mt	145 ± 11.7	127–169
BMI, kg/m^2^	20.1 ± 2.95	17.1–26.0
Waist circumference, cm	68.5 ± 9.45	57.3–85.3
APHV, years	−2.22 ± 1.11	−3.70–−0.30
Physical fitness		
CMJ, cm	19.53 ± 2.98	14.85–24.40
HJ, cm	1.51 ± 0.184	1.14–1.86
TT, seg	12.79 ± 1.09	11.26–15.42
YYIRTI, m	276.67 ± 124.71	120–480

BMI, Body Mass Index; APHV, years at maximum growth velocity; CMJ, countermovement jump; HJ, Horizontal jump; TT, Agility test T-test; YYIRTI, Yo-Yo intermittent endurance test level I.

**Table 2:** Performance in the continuous and fractional SSGs.

	Continuous SSG	Fractional SSG	*p*-Value	ES (95%Cl)	Magnitude
Performance measures during SSG					
CB, n	6.46 ± 3.50	6.33 ± 3,51	0.895	0.04 (−0.53:0.60)	Trivial
RB, n	15.42 ± 3.67	14.96 ± 4.75	0.769	0.09 ( −0.48:0.65)	Trivial
NB, n	9.00 ± 2.86	9.42 ± 4.39	0.774	−0.09 ( −0.65:0.48)	Trivial
LB, n	7.75 ± 2.99	8.25 ± 3.76	0.735	−0.10 ( −0.67:0.47)	Trivial
OB, n	4.17 ± 2.20	5.63 ± 2.85	0.220	−0.38 ( −0.95:0.22)	Small
SS, n	5.67 ± 3.44	6.00 ± 2.60	0.540	−0.18 ( −0.75:0.39)	Trivial
Volume of play, Score	21.9 ± 5.71	21.29 ± 7.61	0.784	0.08 ( −0.49:0.65)	Trivial
Efficiency Index, Score	0.60 ± 0.28	0.64 ± 0.21	0.603	−0.15 ( −0.72:0.42)	Trivial
General Performance, Score	16.97 ± 4.37	21.29 ± 7.61	0.978	−0.01 ( −0.57:0.56)	Trivial
Physiological and psychological response					
PACES, Score	33.73 ± 6.19	33.23 ± 5.75	0.078	0.59 ( −0.06:1.23)	Small
HR, bpm	178.44 ± 8.15	177.27 ± 9.77	0.663	0.13 ( −0.44:0.70)	Trivial
HR, %FCmax	88.51 ± 4.48	88.69 ± 4.88	0.854	−0.05 ( −0.62:0.51)	Trivial

CB: conquered balls; RB: received balls; NB: neutral balls; LB: lost balls; OB: offensive ball; SS: successful shot; PACES: Physical Activity Enjoyment Scale; HR: heart rate; bpm: beats per minute; %HRmax: percentage of maximum heart rate.

**Table 3:** Relationship between anthropometric and physical fitness measures with technical performance measures and enjoyment during small-sided soccer games.

	Technical Performance									Enjoyment
	CB	RB	NB	LB	OB	SS	VJ	EI	GP	PACES
**Continuous small-sided games**										
Anthropometric measurements										
Weight	0.409	−0.150	−0.053	−0.271	0.000	0.091	0.154	0.084	0.409	−0.821 *
Height	0.610 *	−0.006	0.010	−0.275	0.061	0.247	0.370	0.231	0.387	−0.725 *
BMI	0.059	−0.241	−0.172	−0.296	−0.140	−0.033	−0.119	−0.056	−0.114	−0.778 **
WC	0.365	−0.165	−0.142	−0.148	−0.169	0.023	0.118	−0.072	0.031	−0.821 **
APHV	0.27	−0.15	0.02	−0.37	0.22	0.51	0.07	0.53	0.38	−0.062
Physical fitness measures										
CMJ	0.178	0.294	0.429	0.095	0.290	0.217	0.298	0.176	0.308	0.032
HJ	0.556	0.022	0.171	−0.258	0.607 *	0.494	0.355	0.67 *	0.654 *	−0.064
TT	−0.434	0.115	0.195	0.386	−0.305	−0.634 *	−0.192	−0.628 *	−0.524	0.071
YYIRTI	0.404	−0.096	−0.168	0.148	0.241	0.535	0.186	0.408	0.381	0.349
**Fractional small-sided games **										
Anthropometric measurements										
Weight	0.151	0.409	0.237	0.185	0.424	0.303	0.325	0.421	0.414	−0.837 **
Height	0.293	0.380	0.156	0.205	0.490	0.484	0.373	0.578 *	0.514	−0.747 **
BMI	0.063	0.459	0.370	0.246	0.366	0.108	0.316	0.205	0.319	−0.821 **
WC	0.179	0.478	0.224	0.312	0.445	0.216	0.381	0.275	0.397	−0.904 **
APHV	0.11	0.11	−0.22	−0.11	0.23	0.64 *	0.11	0.71 *	0.38	0.010
Physical fitness measurements										
CMJ	0.031	0.236	0.100	0.117	0.096	0.351	0.161	0.186	0.197	0.171
HJ	0.459	0.366	0.492	0.140	0.465	0.604 *	0.441	0.601 *	0.574	−0.015
TT seg	−0.636 *	−0.687 *	−0.557	−0.464	−0.778 **	−0.73 **	−0.72 **	−0.677 *	−0.815	0.116
YYIRT	0.443	0.218	0.116	0.211	0.359	0.515	0.340	0.343	0.394	0.364

CB: conquered balls; RB: received balls; NB: neutral balls; LB: lost balls; OB: offensive ball; SS: successful shots; VJ: volume of play; EI: efficiency index; GP: general performance; WC: waist circumference; BMI, Body Mass Index; APHV, years at maximum height velocity; CMJ, countermovement jump; YYIRTI, Yo-Yo intermittent endurance test level I. * *p* < 0.05; ** *p* < 0.001.

## Data Availability

The underlying research materials related to this paper are available from the corresponding author upon request.
